# Cardiac Tamponade following Mitral Valve Replacement for Active Infective Endocarditis with Ring Abscess

**DOI:** 10.1155/2015/790213

**Published:** 2015-01-22

**Authors:** R. Ranjan, T. Lawrence

**Affiliations:** Department of Cardiology, Cardiovascular and Thoracic Institute, University of Southern California, Los Angeles, CA 90033, USA

## Abstract

Periannular extension and abscess formation are rare but deadly complications of infective endocarditis (IE) with high mortality. Multimodality cardiac imaging, invasive and noninvasive, is needed to accurately define the extent of the disease. Debridement, reconstruction, and valve replacement, often performed in an emergent setting, remain the treatment of choice. Here we present a case of severe IE in a 29-year-old intravenous drug user who after undergoing debridement of the abscess, annular reconstruction, and mitral valve replacement (MVR) presented with recurrence of shortness of breath and pedal edema. Transthoracic echocardiogram (TTE) showed a 6.2 × 5.5 cm cavity, posterior to and communicating with the left ventricle through a 3 cm wide fistulous opening, in proximity of the reconstructed mitral annulus. The patient underwent a redo MVR with patch closure of the fistulous opening, with good clinical outcome. This case highlights the classic TTE findings and the necessity for close follow-up in the perioperative period in patients undergoing surgery for periannular extension of infection. A cardiac magnetic resonance imaging can be considered, preoperatively, in such cases to identify the extent of myocardial involvement and surgical planning.

## 1. Background

Periannular extension of infection in infective endocarditis (IE) is a serious complication with significant mortality even after prompt surgical management. It is an incompletely understood phenomenon and likely develops as a result of direct extension of infection and tissue necrosis [1–5]. Tissue necrosis and pyogenesis may lead to formation of an abscess cavity which under increased pressure is prone to rupture causing fistulae formation [6]. Transesophageal echocardiogram (TEE) is the diagnostic tool of choice for diagnosis of perivalvular involvement but is less sensitive in detecting diseases of mitral valve compared to aortic valve (57% versus 86%) [7, 8]. Cardiac computed tomography (CCT) and cardiac magnetic resonance imaging (MRI) could be used to supplement findings of TEE in such cases and for surgical planning [9, 10]. Debridement of abscess cavity followed by patch closure and valve replacement often performed emergently is mainstay of treatment.

## 2. Case

**Figure 1:**
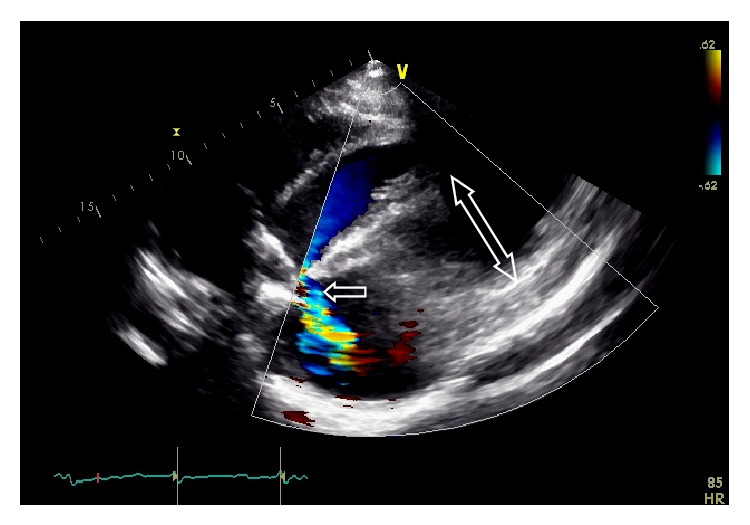
Color Doppler image shows flow from the left ventricle through the fistulous tract (single head arrow) into the pericardial space.

We present a case of a 29-year-old female intravenous drug user who presented to an outside hospital with fever, fatigue, and shortness of breath with exertion. Blood cultures grew oxacillin sensitive* Staphylococcus aureus* (OSSA) and the patient was started on appropriate antibiotics. A transthoracic echocardiogram (TTE) showed vegetation on both leaflets of mitral valve with moderate to severe mitral regurgitation. The patient underwent transesophageal echocardiogram (TEE) that showed vegetation originating from anterior commissar and extending into the mid scallop of the posterior leaflet. A mitral annular abscess was also noted on the TEE. The patient underwent debridement and simple closure of the abscess cavity (1.8 × 1.5 cms) followed by mitral valve replacement (MVR) with a 27 mm St. Jude mechanical valve. Two weeks later the patient was referred to our hospital for lower extremity swelling, fatigue, and altered mental status. Vitals at presentation were BP 115/96 mmHg, heart rate 92 beats/minute, temperature 95.6°F, and respiratory rate of 18/minute. Physical examination revealed bulging neck veins and distant heart sounds. EKG showed low voltage complexes. Leukocyte count was 11,000/*μ*L with predominant neutrophilia. TTE showed communication between the left ventricle and the pericardial cavity through a 3 cm fistulous opening in the region of the posterior mitral annulus. This cavity measured 6.2 × 5.6 cms compared to 1.8 × 1.5 cms prior to the valve replacement (Figure 1). Flow velocity through the fistula was 3.35 m/s with peak gradient of 44 mmHg. There was evidence of diastolic collapse of the right ventricle, interventricular dependence, and exaggerated respiratory variation, all consistent with the diagnosis of cardiac tamponade. TTE also demonstrated periprosthetic insufficiency of mitral valve. Emergent surgery was performed and on opening the pericardium the surgeon was able to clearly see the mechanical valve through the 3 cm wide opening in the posterior aspect of the left ventricular wall. The fistula was repaired with a bovine pericardial patch and a new 25 mm St. Jude mechanical valve was placed at the mitral position. Intraoperative TEE confirmed good valvular function. The patient recovered well and was discharged home a week after the surgery with close follow-up.

## 3. Discussion

Iatrogenic cardiac tamponade (ICT), both acute and subacute, is rare, especially in the era of advanced multimodality cardiac imaging. Most of the case reports in the literature allude to ICT as a complication of either cardiac catheterization or pacemaker insertion. In the pediatric population ICT has been reported as a complication of central venous cannulation and umbilical vein catheterization. In all these cases the precipitating event is left ventricular wall rupture leading to accumulation of blood in the pericardial space. In our patient, the intraoperative TEE performed after initial MVR was normal, as expected. The fistula most likely developed over the next two weeks with necrosis of the infected paravalvular tissue left behind by incomplete debridement. Irrespective of the etiology, the prognosis depends on early diagnosis and prompt surgical repair. A 64-multislice cardiac computed tomography (CT) is superior to TEE in assessment of the degree of paravalvular involvement. In addition, coronary anatomy can also be studied in case concurrent coronary artery bypass is desired [10]. Contrast enhanced cardiac magnetic resonance imaging (CMR) can also be performed for better tissue characterization, noninvasively. After the diagnosis is established and the extent of paravalvular involvement is recognized, debridement and closure of the defect using a Teflon [11] or pericardial patch or adhesion of the ruptured wall to the epicardial surface [12] accompanied by valve replacement is the treatment of choice.

## 4. Follow-Up

The patient was evaluated one month after discharge during a postoperative follow-up visit. The sternal wound was healing well and apart from the pain at the closure site the patient was asymptomatic. The patient also underwent a follow-up transthoracic echocardiogram which showed good valve function and hemodynamics. The care was then transferred to a local cardiologist and a primary care physician.

## 5. Conclusion

This case highlights the importance of multimodality imaging for surgical planning and close postoperative follow-up of patients with paravalvular abscess.
